# Supplementary material for: Monitoring the Interfacial Polymerization of Piperazine and Trimesoyl Chloride with Hydrophilic Interlayer or Macromolecular Additive by In Situ FT-IR Spectroscopy
Source: Membranes (Basel). 2020 Jan 7;10(1):12. doi: 10.3390/membranes10010012 (PMC7022637; doi:10.3390/membranes10010012)
Supplement: Supplementary file 1 [file membranes-10-00012-s001.pdf]

Electronic Supporting Information

Monitoring the Interfacial Polymerization of Piperazine and Trimesoyl Chloride with Hydrophilic Interlayer or Macromolecular Additive by in-situ FT-IR Spectroscopy

Xi Yang [1*]

[1] Department of Polymer Science & Engineering, Zhejiang University, China
* Correspondence: xkyang@zju.edu.cn (11529019@zju.edu.cn)

*Corresponding authors. E-mail:xkyang@zju.edu.cn (Xi Yang); Department of Polymer Science and Engineering, Zhejiang University, Hangzhou 310027, China.

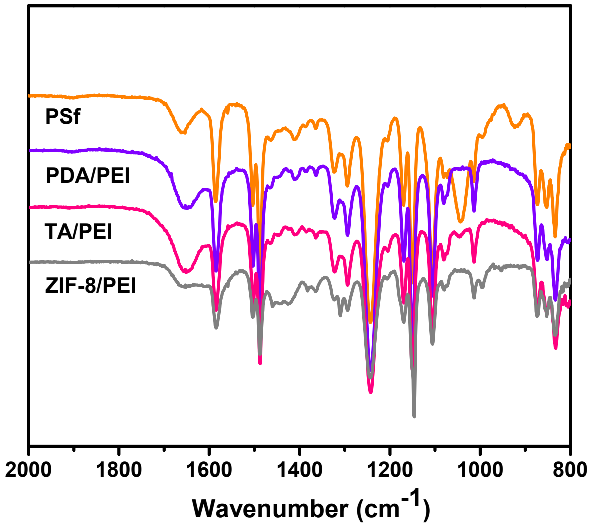

Figure S1. ATR/FT-IR spectra of pristine PSf substrate and PSf substrates modified with PDA/PEI, TA/PEI and ZIF-8/PEI interlayers, respectively.

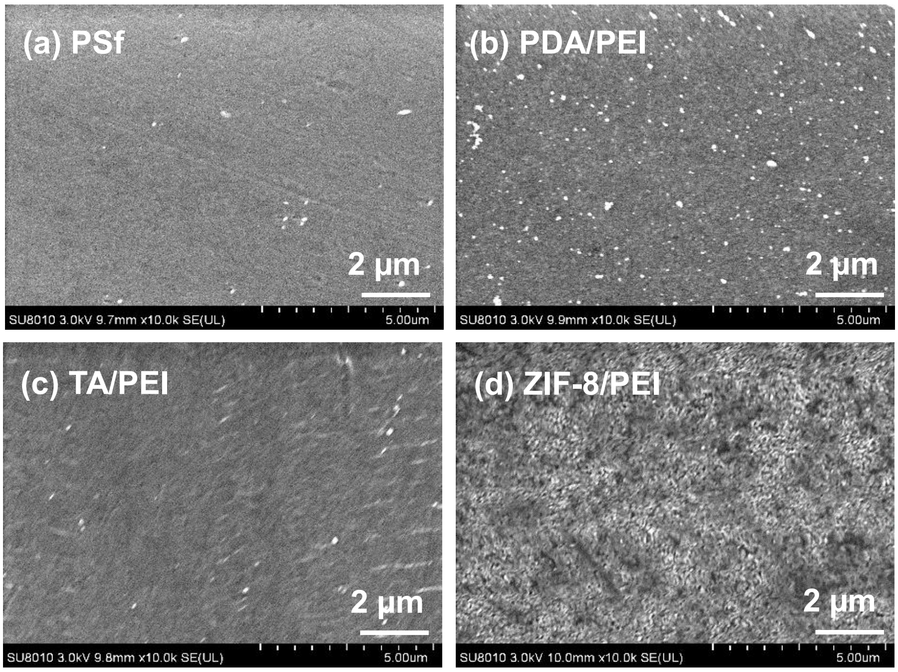

Figure S2. Surface morphologies observed by FESEM images of pristine PSf substrate and PSf substrates modified with PDA/PEI, TA/PEI and ZIF-8/PEI interlayers, respectively.

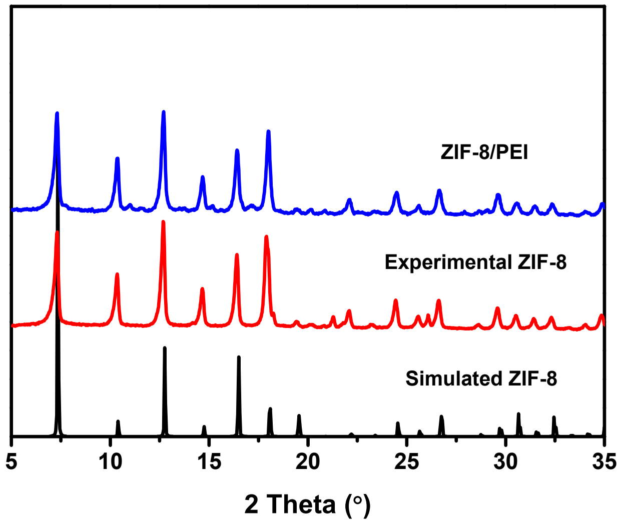

Figure S3. XRD spectra of the synthesized ZIF-8 and ZIF-8/PEI.

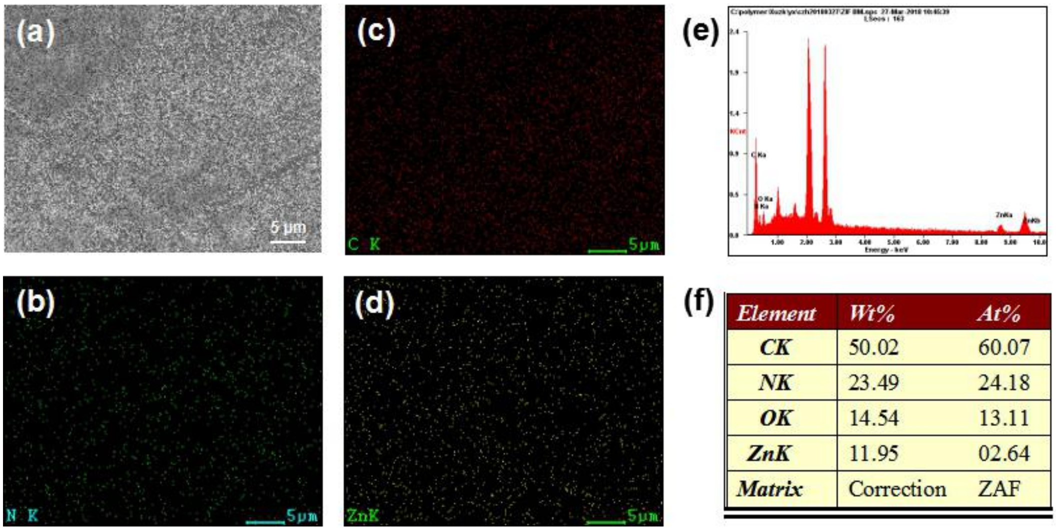

Element	*Wt%*	*At%*
CK	50.02	60.07
NK	23.49	24.18
OK	14.54	13.11
ZnK	11.95	02.64
Matrix	Correction	ZAF

Figure S4. FESEM and EDX images of the ZIF-8/PEI modified PSf substrate.

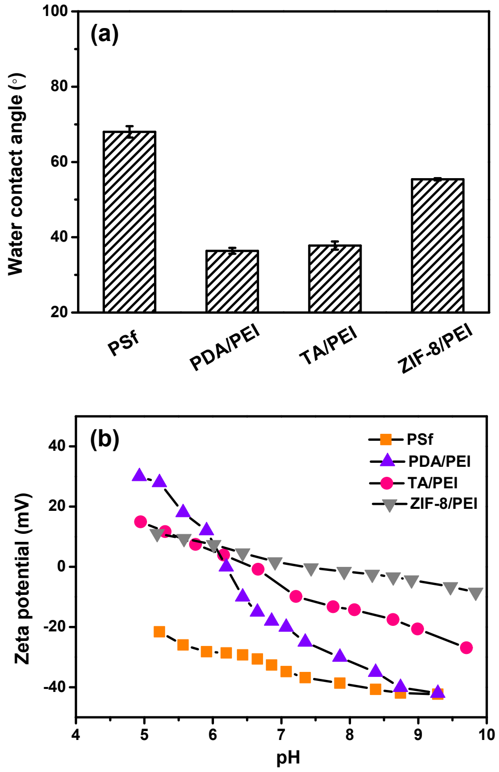

Figure S5. (a) Water contact angle and (b) zeta potential of PSf substrate and PSf substrates modified with PDA/PEI, TA/PEI and ZIF-8/PEI interlayers, respectively.

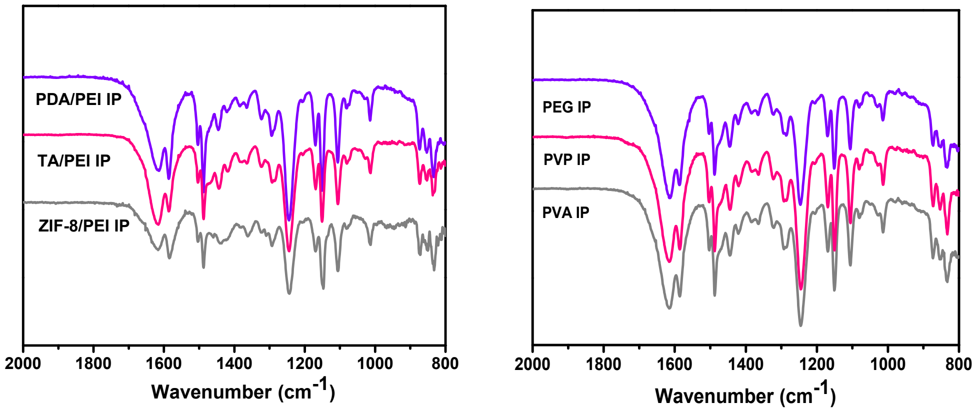

Figure S6. ATR/FT-IR spectra of the polyamide-based membranes formed on the substrates modified with the interlayers on the substrates or with the macromolecular additives in the solution of PIP.

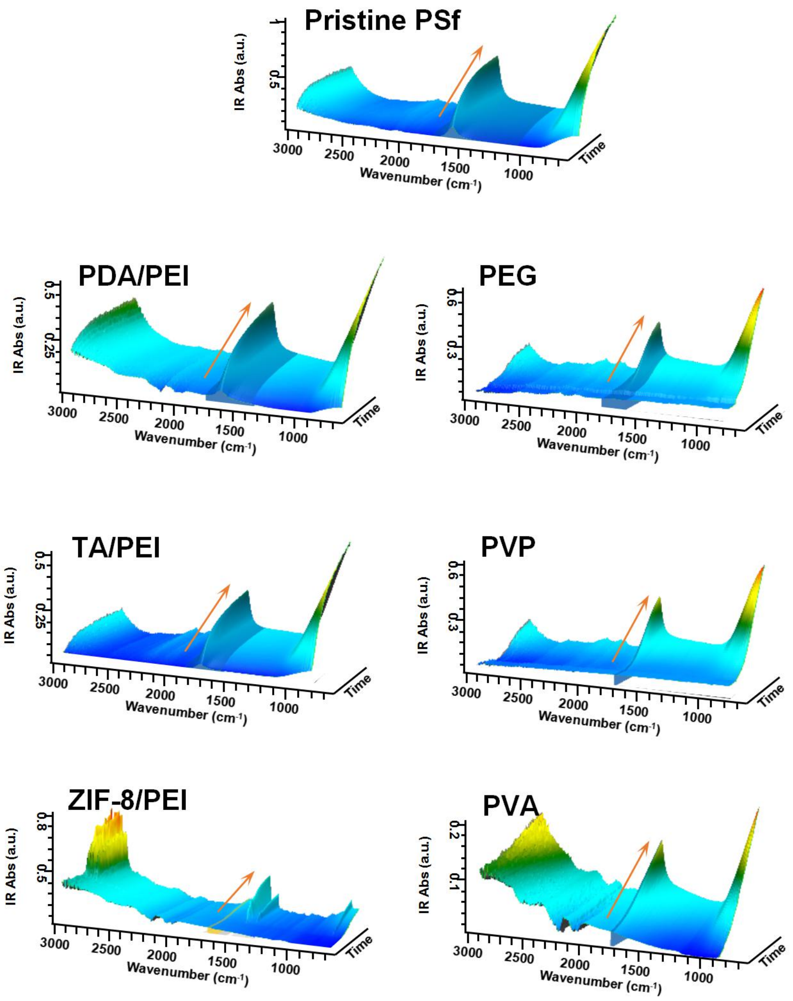

Figure S7. Three-dimension spectra measured by in-situ FT-IR spectroscopy for the polyamide formation as a function of interfacial polymerization time, with different modified interlayers on the substrates or macromolecular additives in the solution of PIP.

UV-vis analysis of diamine diffusivity

The diffusion of diamine monomers were measured by UV-vis absorption spectra, using an ultraviolet spectrophotometer (Shimadzu, UV 2450, Japan). By taking 3 mL hexane solution approximately at the hexane/water interface, diamine concentration and diffusivity were measured by ultraviolet analyses and acyl chloride monomer was not added in the hexane phase for the diffusion time for 10 min. Initial diffusivity D_0 and the corresponding D are calculated by the following equation (Eq S1):

$$J = \frac{dm}{Adt} = -D_0 \left(\frac{\partial C}{\partial X} \right) \quad \text{(S1)}$$

where J is the diamine diffusive flux, dm, A and dt are the diamine mass, contact area and diffusion time, respectively. D_0 is the initial diffusivity, ∂C and ∂X are the concentration change and diffusion distance (approximately ~ 10^{-5} cm).

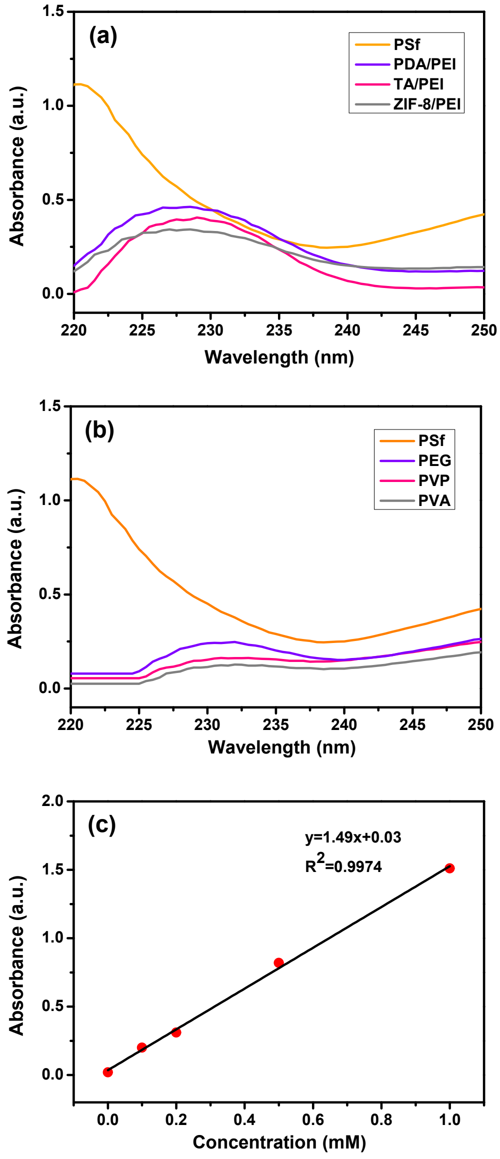

Figure S8. UV-vis absorption spectra of diamine monomer diffusion from water to hexane (the organic solution is hexane without acyl chloride monomer) for the diamine diffusion time of 10 min and the calibration stand curve of absorbance *vs.* diamine concentration.

Table S1. According to UV-vis adsorption spectra, the concentration and diffusivity of diamine monomers were determined, which were calculated by equation (S1).

	PSf	PDA/PEI	TA/PEI	ZIF-8/PEI	PEG	PVP	PVA
Absorbance (a.u.)	1.115	0.459	0.406	0.343	0.248	0.163	0.128
Concentration (mM)	0.728	0.288	0.252	0.210	0.146	0.089	0.066
Diffusivity ($\times 10^{-6}$ cm^2/s)	11.96	4.73	4.14	3.45	2.39	1.46	1.08

Adsorption of the diamine monomers measured by TOC analyzer

The PSf substrate and substrates with the modified interlayers were cut in to square pieces of 1 cm^2 area and immersed in diamine solution for 10 minutes. The equilibrium adsorption amount were obtained after 24 h diamine release in 30 mL DI water and total organic carbon of diamine monomers were quantified by a TOC analyzer (TOC, GE Sievers InnovOx ES, USA).

Table S2. Adsorption mass of diamine monomers measured by TOC analyzer.

	PSf	PDA/PEI	TA/PEI	ZIF-8/PEI	PEG	PVP	PVA
Mass (g)	0.00745	0.00759	0.00768	0.00772	0.00745	0.00745	0.00745
PIP (ppm)	8.78	29.9	88.6	224	9.10	10.2	10.5
Q (mg/g)	1.18	3.94	11.5	29.0	1.22	1.37	1.41

Binding energy simulation

Binding energy simulations were performed between the PIP and interlayers on the substrates or with macromolecular additives in the PIP solution. It is conducted in Materials Studio 7.0. Firstly, all the molecules were constructed and then optimized by Forcite module. The binding energy (E_b) in the COMPASS force-field of PIP and other molecules were calculated using the equation (Eq S2) [Ref S1]:

$$E_b = E_{PIP\text{-}molecule} - (E_{PIP} + E_{molecule}) \quad \text{(S2)}$$

where $E_{PIP\text{-}molecule}$ is the system total energy, E_{PIP} and $E_{molecule}$ are the energies of the PIP and other molecules, respectively. The binding energy is the combination of attractive and repulsive forces between these molecules.

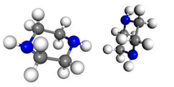

ΔE (PIP-PIP) = -1.51 kcal/mol

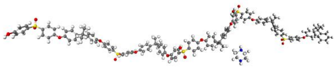

ΔE (PSf-PIP) = -2.06 kcal/mol

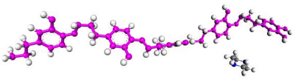

ΔE (PDA-PIP) = -5.29 kcal/mol

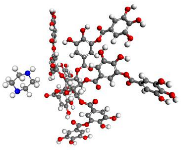

ΔE (TA-PIP) = -6.20 kcal/mol

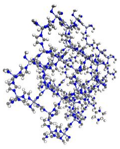

ΔE (ZIF-8-PIP) = -7.06 kcal/mol

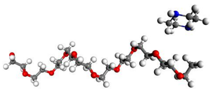

ΔE (PEG-PIP) = -2.65 kcal/mol

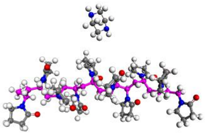

ΔE (PVP-PIP) = -2.99 kcal/mol

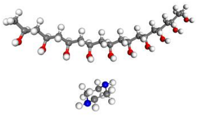

ΔE (PVA-PIP) = -3.43 kcal/mol

Figure S9. Binding energies calculated between PIP and other molecules.

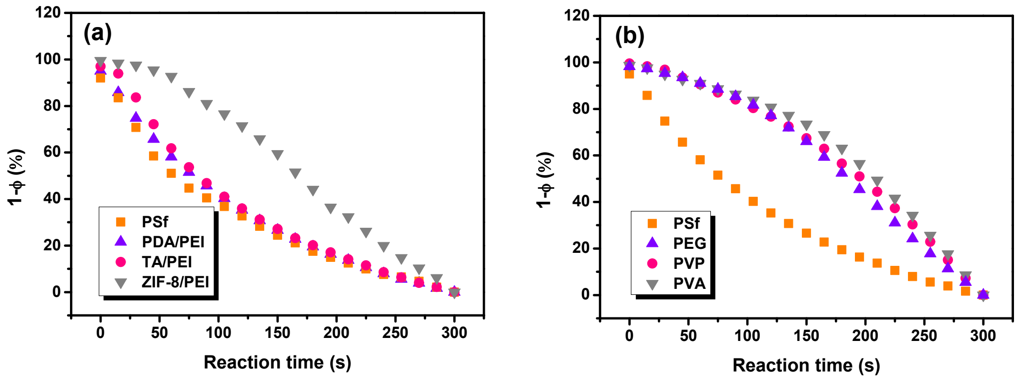

Figure S10. Polyamide volume fractions converted from in-situ FT-IR spectra.

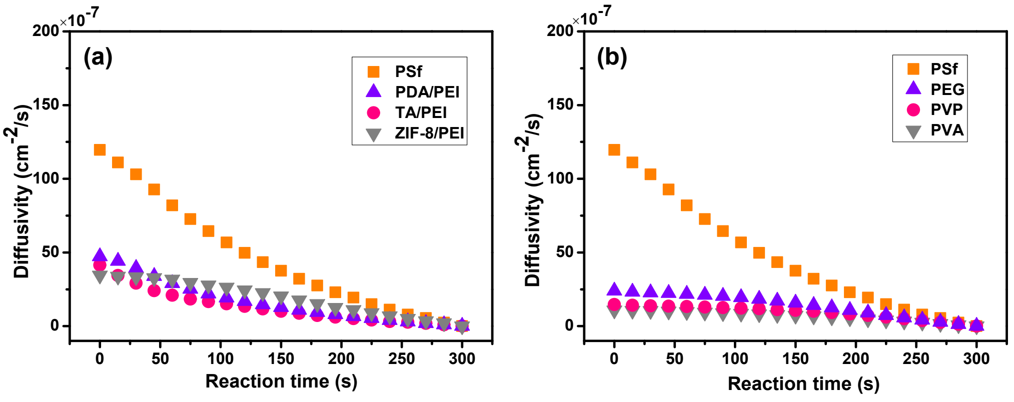

Figure S11. Diamine diffusivity with the interfacial polymerization time, showing depressed and "self-limiting" effect of Eq S3.

$$D = D_0 \left(1 - \phi\right)^{\alpha} \qquad \text{(S3)}$$

where the corresponding diffusivity D is calculated from the empirical formula, in which D_0 is the initial diffusivity, φ is the polyamide volume fraction and α typically varies in the range of $1 < \alpha < 3$ [Ref S2].

Determination of the polyamide layer thickness by FT-IR spectroscopy

Thickness of the polyamide layer was probed by FT-IR with an ATR accessory (Ge crystal, 45° incident angle), using the equations (Eq S4 and S5) [from Ref S3]:

$$d_p = \frac{\lambda}{2\pi\sqrt{n_1^2 \sin^2\theta - n_2^2}} \quad \text{(S4)}$$

where d_p is the penetration depth, λ is the wavelength of infrared radiations, n_1 and n_2 are refractive indices of the crystal and the sample. Since $n_1 = 4.0$ (Ge crystal), $n_2 = 1.50$ (polyamide sample) and θ = 45°, $d_p = 0.066\ \lambda$. The characteristic absorbance at 1640 cm^{-1} was transformed into the polyamide layer thickness [Ref S4]:

$$T = -\ln\left[\frac{A_b(T)}{A_b(0)}\right] \times \frac{d_p}{2} \quad \text{(S5)}$$

where T is the thickness, $A_b(T)$ and $A_b(0)$ are the absorbance of a band at the layer thickness of T and 0, respectively.

Table S3. Polyamide layer thickness acquired from *in-situ* FT-IR spectroscopy, which were fitted mathematically with the equation of $X = At^b$.

	PSf	PDA/PEI	TA/PEI	ZIF-8/PEI	PEG	PVP	PVA
A	24.8	7.3	4.0	0.6	5.4	3.3	0.5
b	1/2	1/2	1/2	1.0	2/3	2/3	1.0

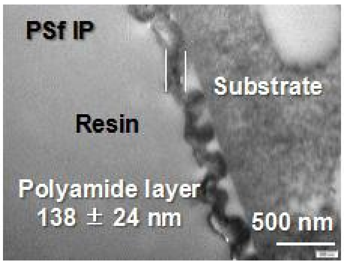

Figure S12. TEM observation of polyamide layer thickness observed on the pristine PSf substrate.

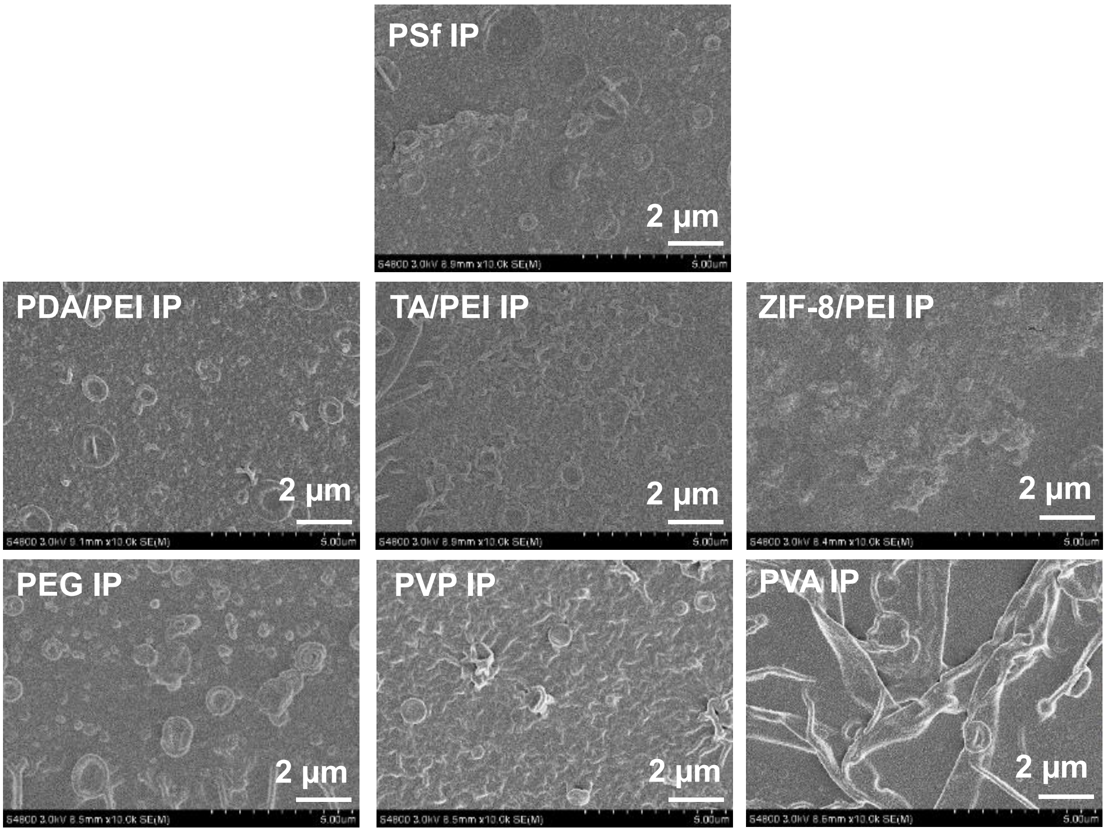

Figure S13. FESEM images of surface morphologies of polyamide layers formed on the PSf substrate and PSf substrates modified with interlayers on the substrates or doped polyamide layer with macromolecular additives in the solution of PIP.

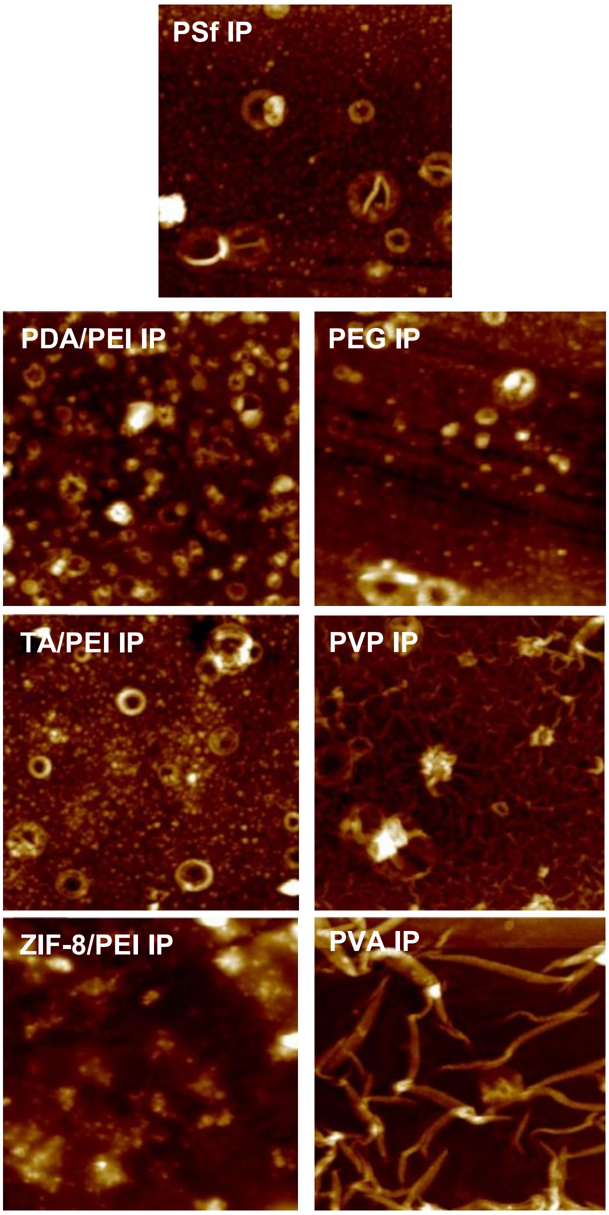

Figure S14. AFM images of surface topographies of polyamide layers formed on the PSf substrate and PSf substrates modified with interlayers on the substrates or doped polyamide layer with macromolecular additives in the solution of PIP.

Table S4. Surface roughness of the membrane samples measured from AFM images.

Membranes	R_a (nm)	R_q (nm)
PSf IP	53	79
PDA/PEI IP	52	81
TA/PEI IP	66	84
ZIF-8/PEI IP	131	189
PEG IP	54	73
PVP IP	72	103
PVA IP	95	113

Table S5. XPS analyses of element component of the polyamide membrane surface and the corresponding calculated O/N ratio.

Membranes	C (%)	N (%)	O (%)	O/N
PSf IP	70.51	12.15	17.34	1.43
PDA/PEI IP	67.72	10.60	21.68	2.04
TA/PEI IP	67.62	10.91	21.47	1.97
ZIF-8/PEI IP	68.38	11.80	19.82	1.68
PEG IP	69.01	11.57	19.42	1.68
PVP IP	68.81	12.12	19.07	1.57
PVA IP	69.65	13.19	17.15	1.30

Table S6. Water contact angle and zeta potential of the formed polyamide membrane.

Membranes	Contact angle (°)	Zeta potential (mV)
PSf IP	75 ± 4.4	-38.2
PDA/PEI IP	35 ± 3.6	-16.8
TA/PEI IP	43 ± 3.3	-13.4
ZIF-8/PEI IP	48 ± 4.2	-12.6
PEG IP	29 ± 3.2	-32.5
PVP IP	28 ± 2.4	-29.4
PVA IP	26 ± 2.8	-25.6

Table S7. Rejections of polyamide membrane for different kinds of inorganic salts.

Membranes	$R_{PDA/PEI}$ (%)	$R_{TA/PEI}$ (%)	$R_{ZIF-8/PEI}$ (%)	R_{PEG} (%)	R_{PVP} (%)	R_{PVA} (%)
Na_2SO_4	97.5	97.8	98.2	97.4	98.2	98.4
$MgSO_4$	97.2	97.6	97.8	97.5	97.8	98.2
$MgCl_2$	69.5	70.3	73.4	80.9	85.6	87.3
$CaCl_2$	68.1	68.6	69.4	78.4	83.6	85.4
NaCl	42.5	43.6	45.2	52.8	53.6	54.2

References

S1. Zhai, Z.; Jiang, C. Fabrication of advanced nanofiltration membranes with nanostrand hybrid morphology mediated by ultrafast noria–polyethyleneimine codeposition. *J. Mater. Chem. A* **2018**, *6*, 21207–21215.

S2. Freger, V. Kinetics of film formation by interfacial polycondensation. *Langmuir* **2005**, *21*, 1884–1894.

S3. Ohta, K.; Iwamoto, R. Experimental proof of the relation between thickness of the probed surface layer and absorbance in FT-IR/ATR spectroscopy. *Appl. Spectrosc.* **1985**, *39*, 418–425.

S4. Singh, P. S.; Joshi, S. V. Probing the structural variations of thin film composite RO membranes obtained by coating polyamide over polysulfone membranes of different pore dimensions. *J. Memb. Sci.* **2006**, *278*, 19–25.
